# Segregated Co-activation Patterns in the Emergence of Decision Confidence During Visual Perception

**DOI:** 10.3389/fnsys.2020.557693

**Published:** 2020-11-10

**Authors:** Cilia Jaeger, Sarah Glim, Cristiana Dimulescu, Anja Ries, Christian Sorg, Afra Wohlschläger

**Affiliations:** ^1^Department of Neuroradiology, Technical University of Munich, Munich, Germany; ^2^TUM-Neuroimaging Center, Technical University of Munich, Munich, Germany; ^3^Graduate School of Systemic Neurosciences, LMU Munich, Planegg-Martinsried, Germany; ^4^Department of Psychiatry, Technical University of Munich, Munich, Germany

**Keywords:** metacognition, decision confidence, fMRI, supplementary eye field, visual perception

## Abstract

Visual metacognition—the introspection and evaluation of one’s own visual perceptual processes—is measured through both decision confidence and “metacognitive efficiency.” Metacognitive efficiency refers to an individual’s ability to accurately judge incorrect and correct decisions through confidence ratings given their task performance. Previous imaging studies in humans and nonhuman primates reported widely distributed brain regions being involved in decision confidence and metacognition. However, the neural correlates of metacognition are remarkably inconsistent across studies concerning spatial outline. Therefore, this study investigates the neural correlates of visual metacognition by examining co-activation across regions that scale with visual decision confidence. We hypothesized that interacting processes of perceptual and metacognitive performance contribute to the arising decision confidence in distributed, but segregable co-activating brain regions. To test this hypothesis, we performed task-fMRI in healthy humans during a visual backward masking task with four-scale, post-decision confidence ratings. We measured blood oxygenation covariation patterns, which served as a physiological proxy for co-activation across brain regions. Decision confidence ratings and an individual’s metacognitive efficiency served as behavioral measures for metacognition. We found three distinct co-activation clusters involved in decision confidence: the first included right-centered fronto-temporal-parietal regions, the second included left temporal and parietal regions, and the left basal forebrain (BF), and the third included cerebellar regions. The right fronto-temporal-parietal cluster including the supplementary eye field and the right basal forebrain showed stronger co-activation in subjects with higher metacognitive efficiency. Our results provide novel evidence for co-activation of widely distributed fronto-parieto-temporal regions involved in visual confidence. The supplementary eye field was the only region that activated for both decision confidence and metacognitive efficiency, suggesting the supplementary eye field plays a key role in visual metacognition. Our results link findings in electrophysiology studies and human fMRI studies and provide evidence that confidence estimates arise from the integration of multiple information processing pathways.

## Introduction

This study investigates the neural correlates of visual metacognition by examining co-activation across regions that vary relative to visual decision confidence. Metacognition is defined as the ability to introspect and evaluate the quality of one’s decision making (Metcalfe and Shimamura, 1994; Shimamura, 2000; Fleming and Lau, 2014). Metacognition is crucial for guiding behavior, especially in the absence of external feedback, and mitigates future mistakes (Metcalfe and Shimamura, 1994; Yeung and Summerfield, 2012). Visual metacognition is commonly operationalized through subjective confidence ratings about the accuracy of one’s visual decision-making processes. Decision confidence has been extensively used to quantify metacognition in humans and primates (Kepecs and Mainen, 2012; Mamassian, 2016; Bang and Fleming, 2018; Vaccaro and Fleming, 2018, for review). Yet, the neural underpinnings of visual metacognition remain unclear due to diverse findings across studies.

Neural correlates of visual metacognition have been reported for widely distributed brain regions for two classes of studies. One class of studies, mostly performing fMRI in humans, primarily identified regions in the prefrontal and cingulo-opercular cortex. Metacognitive ability has been correlated to confidence-related activity in the dorsolateral prefrontal cortex (dlPFC; Lau and Passingham, 2006; Del Cul et al., 2009), rostrolateral prefrontal cortex (Fleming et al., 2012; Morales et al., 2018), dorsal anterior cingulate cortex (dACC; Fleming et al., 2012; Bang and Fleming, 2018; Morales et al., 2018), and the ventral striatum (Hebart et al., 2014). These results are conceptualized by a model that suggests that objective decision making and metacognitive performance, measured by decision confidence, occur in coupled but distinct networks (Pasquali et al., 2010; Grimaldi et al., 2015; Fleming and Daw, 2017). In more detail, visual confidence is thought to emerge in prefrontal and frontal areas and reverberate back through recurrent pathways to parietal and early visual areas (Del Cul et al., 2009; Fleming et al., 2010; Fleming and Dolan, 2012; Fleming and Daw, 2017). Behaviorally, objective task performance and subjective evaluation of perceptual decisions through confidence ratings can be dissociated, as the two processes occur independently (Maniscalco and Lau, 2012; Fleming and Daw, 2017; Qiu et al., 2018). For example, disassociation occurs when task performance is poor, yet the subject reports high confidence of being correct. Another class of studies, mostly performing electrophysiology in humans (Gherman and Philiastides, 2015) and non-human primates associated the lateral inferior parietal lobe (LIP), an area known to be involved in decision making (Kiani and Shadlen, 2009), and the supplementary eye fields (SEF; Middlebrooks and Sommer, 2012; So and Stuphorn, 2015) with visual metacognition. These results are conceptualized by a different model, in which task choice and decision confidence arise from the same internal state (Kiani and Shadlen, 2009; Kiani et al., 2014; Van Den Berg et al., 2016). Sensory evidence accumulates until a perceptual decision threshold for one type of stimulus is reached. During the metacognitive, second-order decision, the amount of confidence is determined by the distance between the decision boundary and additional accumulated sensory evidence for the different choice options (Pleskac and Busemeyer, 2010; Kiani et al., 2014). The question remains whether these distinct regions associated with decision confidence reflect the confidence estimate as one entity, or whether activation in these regions reflect different information subprocesses that contribute to the confidence estimate. Therefore, we are asking the question, whether these distinct sets of brain regions relevant for decision confidence are linked by brain co-activation, which would partially explain incongruent findings. Such a co-activation-focused view on brain activity may help set apart distinct correlates that covary with decision confidence.

Generally, confidence measures serve as good proxies for estimating the degree of decision accuracy in healthy subjects (Kunimoto et al., 2001; Kepecs and Mainen, 2012; Pouget et al., 2016). However, individuals vary in their ability to accurately judge their performance. Overall self-confidence may bias an individual to over- or underestimate discrimination accuracy, which generates a bias in metacognition (Washburn et al., 2005; Maniscalco and Lau, 2012; Fleming and Lau, 2014). For example, subjects experiencing relative blindsight accurately detect and discriminate between visual stimuli, yet they underestimate their task performance and report low confidence ratings (Lau and Passingham, 2006; Silvanto, 2015). The degree by which an observer’s confidence ratings distinguish between incorrect and correct decisions is also confounded by the difficulty of the discrimination task (Maniscalco and Lau, 2012; Fleming and Lau, 2014; Boldt et al., 2017). When measuring visual metacognition, it is important to account for individuals’ task performance. Maniscalco and Lau (2012) developed a measure, called metacognitive efficiency, which measures the accuracy of the metacognitive process itself. More specifically, metacognitive efficiency quantifies a subject’s ability to accurately judge incorrect and correct decisions through confidence ratings given their task performance and reflects intrinsic evaluative processes. Application of the metacognitive efficiency in fMRI studies revealed weak correlations between individual metacognitive efficiency values and BOLD activity in frontal and prefrontal areas as well as left temporal gyri during visual perception (Fleming and Dolan, 2012; McCurdy et al., 2013; Dolan et al., 2018). However, the underlying neural architecture of the association between visual confidence and metacognitive efficiency remains unclear. Therefore, we further ask how the co-activation of confidence correlates with metacognitive efficiency to identify regions that correlate with intrinsic processing of metacognitive judgments independent from task performance.

In the current study, we addressed this question, by implementing a backward-masked visual detection paradigm with post-decision confidence ratings in healthy humans using concurrent task-fMRI. Confidence ratings and metacognitive efficiency values were used as proxies for visual metacognition. Co-varying patterns of blood oxygenation served as a proxy for co-activation of neural regions dependent on decision confidence.

## Materials and Methods

### Participants

Thirty-five healthy, young subjects (24 females, mean age = 25.33, SD = ±3.00) were initially recruited for the study. Testing was stopped pre-emptively in eight subjects who failed to perform above chance during training blocks on the behavioral task. One subject was excluded from the study due to brain abnormalities and two participants were excluded from the analyses because data acquisition could not be completed due to technical problems. Twenty-four subjects completed the study (19 females, mean age = 25.25 years, SD = ±3.19 years) and were included in the analyses. All subjects had a normal or corrected-to-normal vision. Written consent was obtained from all subjects. The study was approved by the in-house ethics review committee at the TUM School of Medicine at the Technical University of Munich.

### Behavioral Task

**Figure 1:**
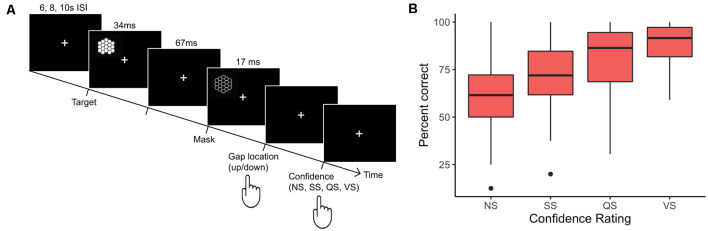
Study trial design and results of the backward masking task for metacognitive confidence. **(A)** The visual backward masking task consisted of a hexagonal honeycomb with a hexagonal gap at either the top or bottom that was presented for 34 ms. A backward mask was presented 67 ms after the target presentation. Subjects had to indicate the gap location and then rate their decision confidence. Subjects were asked to continuously fixate on a fixation cross-present 2 s before and during the task. Targets were presented in the left visual field. **(B)** The distribution of percent correct trials averaged across subject and sessions for the confidence ratings not sure (NS), slightly sure (SS), quite sure (QS), and very sure (VS) are depicted. The horizontal lines indicate the median (NS = 61.54%, SS = 71.94%, QS = 86.36%, and VS = 91.58), the lower and upper hinges of the boxplots represent the first and third quartile, respectively. The lower and upper whisker indicates the minimum and maximum points less than 1.5 times the interquartile range (IQR). Outliers are indicated by dots and are greater than 1.5 × IQR. The mean percent correct for the four confidence ratings were 59.1 ± 13.8% (NS), 73.0 ± 13.1% (SS), 80.9 ± 15.4% (QS), and 88.42 ± 9.4% (VS) respectively. All confidence levels were significantly above chance (*p* < 0.01). VS also was significantly higher than all other confidence levels (*p* < 0.01).

A backward-masked visual perception task (Figure 1A) was adapted from (Wohlschläger et al., 2016; Glim et al., 2020; see also, Haynes et al., 2005) to measure visual confidence. fMRI data were concurrently recorded during the task. Participants fixated on a white cross on black background throughout the experiment. In each trial, the target stimuli were presented for 34 ms in the subjects’ left visual field. The target appeared at a visual angle of 9.7° – 13.4° from the fixation cross in the left visual field. After an interstimulus interval of 67 ms, a color-inverted mask of the target stimuli was presented for 17 ms. The target consisted of an 18-facet hexagonal honeycomb structure with a hexagonal gap either at the top or bottom of the stimuli. Subjects were asked to indicate the location of the gap (top or bottom) with one button press and give a post-decision confidence rating [very sure (VS), quite sure (QS), slightly sure (SS) and not sure (NS)] with a second button press. Key presses were performed on a four-button response box. Asynchronous inter-trial time intervals were randomized between 6.021 ms, 8.028 ms, and 10.035 ms (being multiples of the TR = 2.007 s) with the number of trials per block being 34/100, 33/100, and 33/100, respectively. To control for target position in the hemifield, trials were randomized between two positions in the subjects upper and lower left visual field, respectively. The behavioral task was delivered through the Presentation Software (Neurobehavioral Systems Inc., Berkeley, CA, USA). To familiarize themselves with the task, subjects underwent two training blocks of 50 trials within the MRI-scanner in which subjects had to indicate the gap location. During the first training run, responses were indicated as either correct or incorrect by a green or red signal. Subjects did not have to rate confidence. The second training block was identical to the experimental runs, in which subjects had to give a confidence rating after the target decision. All training blocks were conducted on a separate day outside of the scanner. Testing was stopped for subjects that failed to perform above chance during training blocks. Four experimental blocks of 100 trials per block were pursued.

### Behavioral Outcome Measures

#### Decision Confidence

Decision confidence was directly inferred from the subjects’ second button presses (VS, QS, SS, NS). In all analyses, a response was considered invalid if participants responded before target onset, responded within 200 ms of target presentation, did not respond, or gave more than two answers. Response times were not restricted as subjects were asked to respond as accurately as possible. All trials containing eye movements, blinks, or artifacts within 200 ms of target onset were excluded from the analysis (see Supplementary Methods for identification of eye blinks). To validate the feasibility of the paradigm, the behavioral data had to fulfill two criteria: (i) Correct answers on the target property should be significantly above chance level. Using a two-tailed one-sample Student’s *t*-test, objective task performance was tested for significance above chance level by comparing the number of correct to the total number of trials. (ii) Correctness should significantly increase with an increasing level of confidence. Trials were grouped by level of confidence rating (VS, QS, SS, NS). A larger proportion of correct trials should correspond to higher confidence ratings and task performance near chance should be associated with low confidence ratings. Again, a two-tailed one-sample Student’s *t-test* was performed now for each confidence level. A multiway ANOVA including confidence rating, subjects, and fMRI session as factors and correctness of responses as the dependent variable was also performed across confidence levels, followed by subsequent two-tailed paired-sample Student’s *t*-test between each confidence level. A significant increase in correct responses scaling with confidence conveys valid judgments.

#### Metacognitive Efficiency

To evaluate how well subjects were able to use confidence ratings to accurately judge their task performance, an individual’s metacognitive efficiency was calculated across the entire behavioral task. Task discrimination sensitivity (d’), metacognitive sensitivity (meta-d’), and metacognitive efficiency (meta-d’/d’) were calculated as described by Maniscalco and Lau (2012). In brief, the decision process is separated into two levels: a first-order performance, which reflects how well the task could be solved (correct vs. incorrect responses), and a second-order performance which reflects the correctness of the subject’s judgment on its first-order performance. Therefore, the absolute metacognitive sensitivity value (meta-d’) measures how much information about task performance is used to make a confidence rating. This measure is the ratio between observed metacognitive sensitivity (derived from an individual’s decision confidence ratings across all trials) and expected metacognitive sensitivity, which estimates ideal confidence performance based on task performance. However, the meta-d’ measure is dependent on the quality of first-order performance, quantified by the discrimination sensitivity (d’). The ratio between the discrimination sensitivity (d’) and estimated meta-d’ can then be calculated to obtain metacognitive sensitivity value independent of response bias or task performance. The ratio (meta-d’/d’) is defined as metacognitive efficiency and measures the accuracy of the metacognitive process independent from the perceptual process (see Maniscalco and Lau, 2012, or http://www.columbia.edu/bsm2105/type2sdt/archive/index.html for more detailed information). We used the metacognitive efficiency ratio in our experiment to identify how much decision confidence conveyed a subject’s metacognitive performance independent from task performance. Furthermore, a Pearson correlation between meta-d’/d’ and d’ was also performed to ensure that indeed these measures were independent of each other in our data and described distinguishable processes.

### Imaging Data Acquisition and Processing

#### Imaging Data Acquisition

Imaging data were acquired on a Phillips Ingenia 3T during two appointments. Appointment 1 included a structural MRI scan (MPRAGE, TE = 4 ms, TR = 9 ms, flip angle = 8°, FoV = 240 × 240 mm, 340 slices, voxel size = 0.5 × 0.5 × 0.5 mm). Appointment 2 was focused on the fMRI scans of the masking paradigm. The fixation of the subjects was assessed from concurrently acquired electro-oculography EOG (see Supplementary Methods for more detail). Four-hundred-and-two whole-brain echo-planar imaging (EPI) scans were acquired for each of the four runs (TE = 30 ms, TR = 2.007 s, flip angle = 80°, FoV = 192 × 192 mm, voxel size = 3 × 3 × 3 mm, 36 slices, slice thickness = 3 mm, no interslice gap).

#### Imaging Data Processing

All fMRI data of the masking paradigm was processed and analyzed using statistical parametric mapping (SPM12^1^) and customized Matlab (MATLAB 2016b, The MathWorks, Inc., Natick, MA, USA) scripts. Slices were slice-time corrected, realigned, and unwarped. The functional images were standardized to anatomical space by co-registering the T1-anatomical scan for each subject. The structural images co-registered to the functional images were segmented and spatially normalized to the ICBM space template of European brains. Functional images were normalized with identical transformations. Images were smoothened with an 8 mm Gaussian Kernel.

### Imaging Data Analysis

#### Voxel-Wise Activation Analysis With Decision Confidence, General Linear Model Analysis

A two-stage, mixed-effect analysis was performed in SPM12. A single-subject, fixed effect model was implemented on the first-level using a GLM approach. To model any correlation between confidence and increase in BOLD responses, decision confidence levels were modeled in parametric regressors (paramDC) including only correct trials. Blood-oxygen-level dependent (BOLD) event-related responses were modeled as stick functions convolved with two basic functions in separate regressors: the canonical hemodynamic response function (HRF) and the first derivative in time (TD) of the canonical HRF, to account for possible differences in BOLD response timing. The temporal derivative of the canonical HRF by its shape is better suited to fit an early rise of the BOLD signal after a stimulus. In the following, a strong weight attached to this regressor by the GLM is interpreted as a strong early response. Movement parameters as well as incorrect responses (including eye movement and blinks) were modeled as nuisance regressors. On the second level, contrasts relating to the parametric modulation of BOLD activity dependent on confidence were compared across subjects for statistical significance in one-sample *t*-tests for the canonical HRF and the TD HRF, respectively. Clusters were regarded as significant at a threshold of *p*_FWE_ < 0.05 family-wise error-corrected for multiple comparisons. The location of activation maps was determined with the Anatomy Toolbox (Eickhoff et al., 2005) within SPM.

#### Voxel-Wise Activation Analysis With Metacognitive Efficiency, General Linear Model Analysis

On the second level, contrasts relating to the parametric modulation of canonical HRF and TD HRF were related to the metacognitive efficiency value for each subject, which was entered as a covariate into separate GLM analyses, respectively. Effects at cluster-level correction *p*_cc_ < 0.05 with underlying voxel-level of *p*_unc_ < 0.001, uncorrected for multiple comparisons were regarded as significant.

#### Co-activation Analysis of Decision Confidence, Cluster Analysis of Blood Oxygenation Covariation Patterns

In a second analysis, we focused on those regions of interest (ROIs) displaying a significant correlation with decision confidence. For these regions, defined as significant clusters with voxel numbers above 10 from the respective GLM analysis, we defined masks and extracted the within mask mean contrast values per subject of the contrast “paramDC_TD”. This contrast quantifies the amount of parametric dependence of the early BOLD response on decision confidence. A spherical ROI (8 mm radius) within the pgACC (MNI: *x/y/z* = −2/44/10), which has previously been shown to be involved in decision confidence (Bang and Fleming, 2018), was included. Within a given ROI, the variation of BOLD activity increase with decision confidence across subjects was quantified in a vector. Subsequent cluster analysis across ROI-vectors groups those assemblies of ROIs varying similarly across subjects and separated those ROIs varying independently across subjects. Cluster analysis was realized as *k*-means clustering with *k* = 3 and 50 repetitions. A silhouette analysis was performed to assess the attribution of each ROI to the resulting ROI-clusters. A value of *K* = 3 robustly produced identical clusters while higher values of *K* did not. Cluster centroids were subsequently correlated with the subjects’ metacognitive efficiency values in Pearson correlations to indicate in which cluster the magnitude of the BOLD signal increasing with confidence correlated with metacognitive efficiency. Correlations were regarded as significant at *p* < 0.05, Bonferroni corrected for the number of clusters.

ROI-clusters were visualized in two ways based on Fisher-Z transformed correlation coefficients (FZCC) between ROIs across subjects. In more detail, two ROIs from within the same detected ROI-cluster show enhanced correlation (larger FZCC) of BOLD activity increase with decision confidence across subjects, vs. other ROI pairings. (i) These FZCC between any two ROIs placed on a glass brain were visualized through BrainNet Viewer (Xia et al., 2013) thresholded at 0.5. The line thickness of edges between two regions scale with FZCC. (ii) Alternatively, a visualization *via* multi-dimensional scaling is provided. ROIs are displayed as nodes, FZCCs as edges. In this representation, nodes are close to each other if they have a high correlation. It needs to be noted, that generally multi-dimensional scaling projection into the 2D image plane leads to distortions, so only a general pattern is retained. In our display, node sizes scale with degree centrality, i.e., the average FZCC of one node vs. all others. In both representations, clusters are color-coded.

## Results

### Decision Confidence Behavioral Outcomes

Decision confidence was computed by determining the proportion of correct trials per confidence rating. All of the behavioral statistics were computed by comparing trials of interest to the total number of valid trials. A one-sample *t*-test (*T*_(24)_ = 11.49, *p* < 0.001) showed that the mean percent of correct trials were 77.2% (SD ± 18.7%) and were significantly above chance (corresponding to a mean of 50.0%). The percent of correct to valid trials for each confidence level was: very sure (VS) 88.42 ± 9.4%, quite sure (QS) 80.9 ± 15.4%, slightly sure (SS) 73.0 ± 13.1%, and not sure (NS) 59.1 ± 13.8%. One-sample *t*-tests demonstrating responses were significantly above chance for each confidence level (VS: *T*_(24)_ = 18.79, *p* < 0.001; QS: *T*_(24)_ = 9.83, *p* < 0.001; SS: *T*_(24)_ = 8.62, *p* < 0.001; NS: *T*_(24)_ = 3.23, *p* = 0.002). A multiway ANOVA including confidence rating, subjects, and fMRI session as factors showed the percent of correct trials across confidence levels was statistically significantly above chance (*F*_(1,238)_ = 94.77, *p* < 0.0001). A significant interaction (*F*_(23,238)_ = 3.7, *P* < 0.0001) was evident between confidence rating and subject indicating that confidence ratings varied significantly across subjects (Figure 1B, Supplementary Figure 1). Furthermore, paired *t*-tests were conducted between each confidence level across subjects. A significant increase in mean correct trials was evident with increasing confidence (VS vs. QS, *p* = 0.029; VS vs. SS, *p* < 0.0001; VS vs. NS, *p* < 0.0001; QS vs. SS, *p* = 0.0012, QS vs. NS, *p* < 0.0001; SS vs. NS, *p* < 0.0001, multiple comparison corrected for six comparisons across confidence pairs). The number of trials for each condition averaged across subjects were: VS = 58.13 (SE ± 10.84), QS = 95.42 (SE ± 8.45), SS = 88.29 (SE ± 7.72), NS = 55.96 (SE ± 8.14). Individual overall confidence responses varied with some subjects responding more conservatively and others more liberally (Figure 1B, Supplementary Figure 1). Four participants responded more conservatively, rating no trials with a very sure response, despite perceiving the target correctly. Since confidence was modeled parametrically and we were interested in looking at confidence variability, the subjects were not excluded from the analysis. No difference in significance in behavioral outcomes was observed when performing a secondary decision confidence behavioral analysis, which excluded the four conservative subjects from the analysis.

### Metacognitive Efficiency Behavioral Outcomes

To account for inter-subject variability in task discrimination performance, a ratio between metacognitive sensitivity and discrimination sensitivity (meta-d’/d’) was calculated to measure how efficient trial-by-trial confidence ratings reflect subjects’ ability to accurately judge their performance relative to their type one response (Maniscalco and Lau, 2012; Fleming, 2017). This ratio is called metacognitive efficiency and measures the accuracy of the metacognitive process. Stimulus discrimination sensitivity (d’) and metacognitive sensitivity (meta-d’) were calculated for each subject to compute the metacognitive efficiency ratio (see Maniscalco and Lau, 2012, for methods). Ideal metacognitive efficiency is achieved at a value of 1. A ratio below one suggests that only a portion of the sensory evidence was available for the metacognitive judgment due to sensory signal decay of accumulated noise. Therefore, the confidence rating less accurately predicts task performance accuracy. Whereas a ratio above one indicates subjects perform poorly on task discrimination, yet they are aware of their poor performance and can discriminate accordingly (Maniscalco and Lau, 2012; Fleming and Lau, 2014; Fleming and Daw, 2017). In this study, the metacognitive efficiency ratio was used to account for subject variability in task performance and the subject’s overall confidence in the decision confidence measurements to evaluate unconfounded metacognitive performance. Subjects metacognitive efficiency averaged at 0.79 (range: 0.1–1.9; Supplementary Figure 1). A Pearson correlation between d’ and meta-d’/d’ was performed to further verify that metacognitive efficiency was not biased by task performance. No significant correlation was observed (*r*_(24)_ = −0.3949, *p* = 0.056). The variability in the metacognitive efficiency ratio was then used to correlate inter-subject variability in BOLD activity to metacognitive ability.

### Brain Regions Displaying Significant Activation Dependent on Increasing Decision Confidence

**Figure 2:**
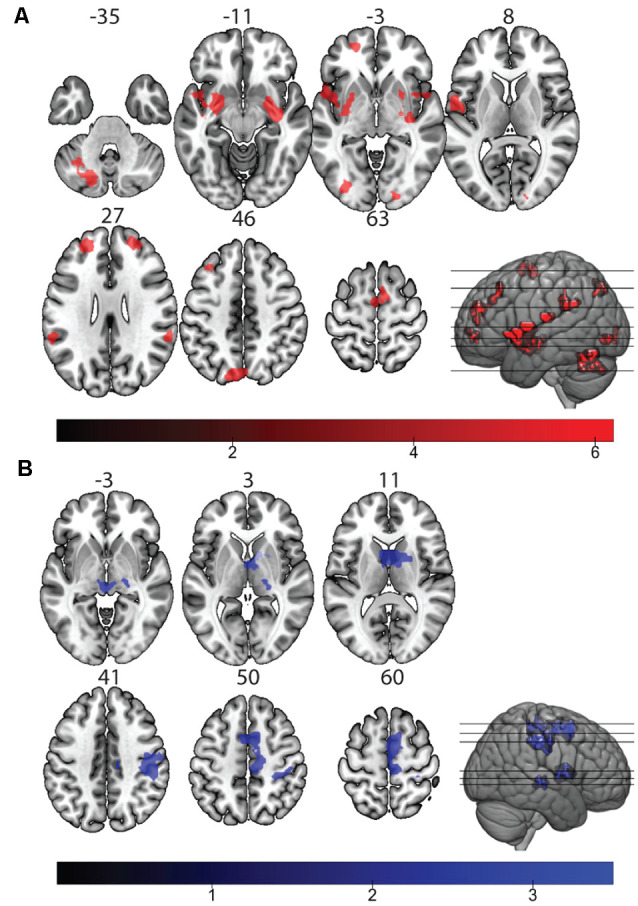
Neural correlates of visual decision confidence and metacognitive efficiency. **(A)** Significant activity of parametrically increasing decision confidence modeled with the blood-oxygen-level dependent (BOLD) temporal derivative of the canonical hemodynamic response function (HRF; Significance level: *p*_FWE_ < 0.05). Significant clusters (red) are bilaterally located in the basal forebrain (BF), supplementary eye fields (SEF), inferior parietal lobe (IPL), supramarginal gyrus, mid frontal gyrus, and mid occipital gyrus (MOG). **(B)** Significant activity of individuals’ metacognitive efficiency correlated with parametrically increasing confidence modeled with a BOLD temporal derivative of the canonical HRF (significance: *p*_cc_ < 0.05 at the *p*_unc_ < 0.001 voxel threshold). Significant clusters (blue) are located in the SEF, putamen, visual thalamus, and right postcentral gyrus.

A mixed-effects factorial design, with increasing confidence modeled parametrically, was used to assess differential activity correlating to degree of decision confidence. Significant activity scaling with confidence was observed in the left inferior parietal lobe (IPL), left anterior cingulate cortex (ACC), left middle frontal gyrus, caudate nucleus, and the mid orbital gyrus at a cluster-level corrected threshold of *p*_cc_ < 0.05 (underlying voxel-level threshold of *p*_unc_ < 0.001, uncorrected; Supplementary Figure 2, Supplementary Table 1). No clusters survived at the family-wise-error-corrected voxel-level when modeled with the canonical HRF. We predicted that increasing confidence may not only result in increased amplitude of BOLD signal across confidence ratings but also result in a faster onset of the peak BOLD response. Incorporating the temporal derivative of the canonical HRF into the analysis allows for the detection of these fast hemodynamic responses. To also capture shifts in the onset of peak BOLD signal, we incorporated the temporal derivative of the canonical HRF to account for peak responses that slightly deviated in time (up to 1 s) from the canonical response curve (Friston et al., 1998; Henson et al., 2002). By far, the largest voxel clusters significantly scaling with confidence were found in the SEF (MNI: *x/y/z* = 6/2/65) and bilateral nuclei Ch4 of the basal forebrain (BF; MNI: *x/y/z* = 27/−4/13 and *x/y/z* = 27/−4/−10) at a timing before the expected canonical hemodynamic responses (Figure 2). Other areas showing a parametric dependence in this early phase of the hemodynamic response at *p*_FWE_ < 0.05 were left parietal-temporal regions, right parietal areas, bilateral mid frontal gyrus, and Rolandic operculum (Table 1).

**Table 1:** Peak voxels of significant clusters for the temporal derivative of the canonical hemodynamic response function (HRF) contrast (*p*_FWE_ < 0.05) with increasing confidence.

Region	MNI coordinates	Voxel-level *z*-score
R SEF	6/2/65	6.69
R BF	27/−4/−10	6.54
L BF	−27/−4/−13	6.30
L superior parietal lobe	−15/−82/47	6.08
R mid frontal gyrus	24/50/26	5.95
L mid occipital gyrus	−33/−82/−4	5.84
L cerebellum	−18/−76/−31	5.80
R mid temporal gyrus	57/−25/−10	5.78
L mid frontal gyrus	−18/47/26	5.73
L frontopolar cortex	−18/56/5	5.53
L rolandic operculum	−57/−1/8	5.50
L supramarginal gyrus	−63/−43/29	5.48
R supramarginal gyrus	63/−43/26	5.46
R cerebellum	27/−61/−34	5.41
L inferior parietal lobe	−60/−22/17	5.40
R V1	18/−91/−1	5.32
R rolandic operculum	51/5/−1	5.23
R mid occipital gyrus	36/−82/−4	5.10
R inferior parietal lobe	63/−16/14	5.07

### BOLD Activity Corresponding to Individual Differences in Metacognitive Efficiency

To isolate metacognitive performance from task performance biases, a measure of metacognitive efficiency (meta-d’/d’) was calculated across the entire study for each subject. An analysis correlating subjects’ metacognitive efficiency with BOLD activity showed that increased metacognitive efficiency is associated with stronger dependence of activity on confidence modeled with the canonical HRF and its temporal derivative in clusters including the SEF, putamen, visual thalamus, and right postcentral gyrus (*p*_cc_ < 0.05, cluster-level corrected on an underlying voxel-threshold of *p*_unc_ < 0.001, Figure 2, Table 2). Activity associated with increased confidence modeled with the canonical HRF contrast also correlated to increased metacognitive efficiency at the same threshold level in the bilateral inferior temporal lobe (*p*_cc_ < 0.05 at *p*_unc_ < 0.001, Supplementary Figure 2, Supplementary Table 2). Notably, activity in the SEF scales with both decision confidence and metacognitive efficiency.

**Table 2:** Peak voxels of significant clusters for the temporal derivative of the canonical HRF contrast with increasing confidence correlating to an increase in individuals’ metacognitive efficiency (*p*_cc_ < 0.05 at the *p*_unc_ < 0.001 voxel threshold).

Region	MNI coordinates	*Z*-score
R Post central gyrus	45/−25/41	4.68
R SEF	3/2/59	4.62
L putamen	−6/2/14	4.51
R visual thalamus	21/−25/2	3.81

### Co-activation of Regions Scaling With Decision Confidence Across Subjects

**Figure 3:**
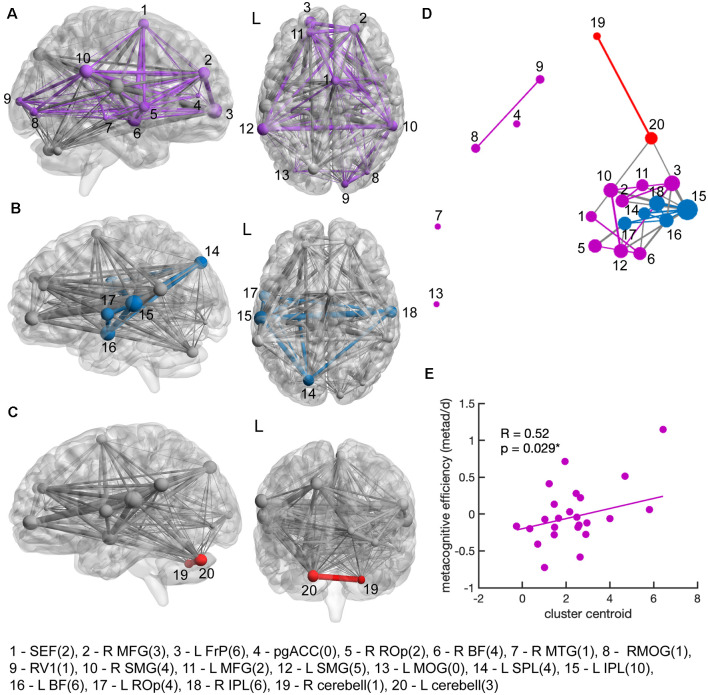
Co-activation clusters relevant to decision confidence and metacognitive efficiency. Co-activation of decision confidence was analyzed across subjects between voxel clusters significantly active at *p*_FWE_ < 0.05 with increasing confidence in the early hemodynamic response phase [modeled with the derivative in time (TD) contrast]. The region pgACC (MNI: −2/44/10) was added, as it has been associated with decision confidence (Bang and Fleming, 2018). Panels **(A–C)** show the locations of the three clusters with activity similarly covarying with confidence in MNI space. **(A)** The largest co-activation cluster (purple) was right-lateralized and included many frontoparietal regions: SEF, right BF, right rolandic operculum (ROp), bilateral midfrontal gyrus (MFG), left frontopolar cortex (FrP), right supramarginal gyrus (SMG), right mid temporal gyrus (MTG), as well as right V1 and bilateral mid occipital gyrus (MOG). **(B)** A second co-activation cluster (blue) was centered in the left temporal parietal region including the: left ROp, bilateral inferior parietal lobe (IPL), left superior parietal lobe (SPL) and left BF. **(C)** The third co-activation cluster (red) was centered in the cerebellum (cerebell). Edges as displayed were constructed from linear correlational analyses of the amount of dependence on confidence within the regions of interest (ROIs) across subjects. Edge size scale with higher correlation coefficients starting at a threshold of *R* > 0.5. Colored edges indicate the correlation degree within a co-activation cluster. Gray edges indicate the strength of the correlation coefficient between two activated regions obtained from the fMRI contrast maps. Node size indicates the degree of centrality. Clusters were visualized with the BrainNet Viewer (Xia et al., 2013, http://www.nitrc.org/projects/bnv/). **(D)** Multidimensional scaling onto a 2D space indicates the degree of co-activation of regions by spatial proximity. Reduction to 2D space leads to inaccuracies but gives a rough illustration of co-activation strength between ROIs. Degree centrality. i.e., the number of edges per node is noted in parentheses within the region labeling below the figure. **(E)** Only the right-centered co-activation cluster (purple cluster) scaled significantly with individual metacognitive efficiency (*R* = 0.52, *p*_(c)_ < 0.005, corrected for multiple comparisons). The line indicates a regression between cluster centroids and metacognitive efficiency values (meta-d’/d’).

We further investigated if co-activation between ROIs dependent on the degree of confidence in the early phase of the BOLD signal separated into segregable processes. Here, parametric modulation of the magnitude of the BOLD signal was used to assess co-activation with confidence across ROIs. The magnitude of the BOLD signal was quantified from the parameter estimates of the regressors parametric in confidence. Parameter estimates were obtained from regions significantly activated for parametric confidence modeled with the temporal derivatives of the canonical HRF contrast (*p*_FWE_ < 0.5) from each subject. Co-activation with confidence was assessed through a correlational analysis between activated ROIs. The underlying model formulates that a high correlation between parameter estimates of two activated ROIs indicates BOLD activity dependent on subjective confidence ratings is linked in both regions and thus suggesting a common process between the two regions. A low correlation coefficient indicates that the activity of region A changes relative to increasing confidence, however, this dependence was unrelated to the dependence on confidence in region B, and thus resulted from a different process. A spherical region around pgACC (MNI: *x/y/z* = −2/44/10) from Bang and Fleming (2018) was included, which is associated with inter-subject decision confidence variability. Figure 3 shows a visualization of the resulting connectivity between ROIs, and the attribution to three stable clusters (see silhouette analysis; Supplementary Figure 3), dissociating separable sets of confidence co-activation clusters: (1) a cluster centered in the right hemisphere (Figure 3A) involving the right fronto-temporal-parietal including bilateral frontopolar regions, mid frontal gyrus, supramarginal gyrus, SEF, and right basal forebrain; (2) a cluster centered in the left parieto-temporal hemisphere (Figure 3B) including the left Rolandic operculum, bilateral inferior parietal lobe (IPL), left superior parietal lobe (SPL) and left basal forebrain; and (3) a cerebellar cluster (Figure 3C). Interestingly, only in co-activation cluster 1 (purple cluster, Figures 3A,E) did signal magnitude increasing with confidence correlate with individual metacognitive efficiency (*R* = 0.52, *p* = 0.029).

## Discussion

In this study, we were first to investigate co-activation patterns of BOLD activity associated with visual confidence and individuals’ metacognitive efficiency during a backward-masked visual detection task with post-decision confidence ratings. By assessing the parametric dependence of confidence in the early phase of the hemodynamic response, we observed the strongest activation significantly scaling with increasing confidence in the supplementary eye field, bilaterally in the basal forebrain, and bilaterally in the parietal cortex and prefrontal cortex. To disentangle how the encoding of decision confidence gives rise to metacognitive performance, independent of task performance and response bias, we correlated individuals’ overall metacognitive efficiency with confidence-dependent BOLD activity. The supplementary eye field, and activated region in the left postcentral gyrus expanding into the inferior parietal lobe, and activated regions in the striatum correlated with increased metacognitive efficiency. Notably, the supplementary eye field was the only region, whose activation correlated with decision confidence and metacognitive efficiency.

We then investigated how the emergence of confidence across distributed regions can be segregated into distinct co-activation processes varying with increasing decision with and correlated these co-activation clusters with individuals’ metacognitive efficiency. We found decision confidence scaled in three segregable co-activation clusters: a right-centered fronto-parieto-temporal (Figure 3A), a left parieto-temporal (Figure 3B), and a cerebellar cluster (Figure 3C). In the right-centered cluster, the magnitude of BOLD signal dependent on confidence correlated strongly between the bilateral supramarginal gyrus, the bilateral mid frontal gyrus, and left frontopolar regions, the SEF, and the right basal forebrain. In the left-centered co-activation cluster, signal magnitude relative to decision confidence correlated between the left inferior parietal lobe, the left basal forebrain, left superior parietal lobe, left Rolandic operculum, and the right inferior parietal lobe. Only the right-centered confidence co-activation cluster including the supplementary eye field scaled significantly with regions significant for individual metacognitive efficiency. This suggests that confidence arises in the cerebellum and two wide-scale networks in the brain. Yet metacognition, measured by the accuracy of judging one’s own perceptual performance through confidence, may be encoded in the supplementary eye field through the emergence of decision confidence in right fronto-temporal-parietal regions.

Although metacognition is crucial in many cognitive processes, such as learning (Elwin et al., 2007; Folke et al., 2017), error monitoring (Boldt and Yeung, 2015; Fitzgerald et al., 2017, and decision making (Fleming, 2016; Van Den Berg et al., 2016; Qiu et al., 2018), it remains debated whether metacognition measured through decision confidence emerges from the same processing mechanism used to make the first-order, task-related decision (Pleskac and Busemeyer, 2010; Kiani et al., 2014; Van Den Berg et al., 2016) or whether second-order, metacognitive decisions occur independently of first-order decisions (Maniscalco and Lau, 2012, 2016; Fleming and Daw, 2017). A divide in the literature has associated parietal and oculomotor decision-making areas with the first theory and prefrontal and frontal executive areas with the latter (Grimaldi et al., 2015). However, the dispute over decision confidence may be explained when examining decision confidence as an entity that arises from the integration of multiple components (Pouget et al., 2016; Bang and Fleming, 2018). During task discrimination, confidence is generally described as a probabilistic computation of a decision being correct given available evidence (Pouget et al., 2016). How different types of evidence are integrated to form a probabilistic outcome remains debated. Our results show that the covariation of activity across three distinct clusters of brain regions becomes more robust with increasing confidence. We speculate that the co-variation of confidence within these three segregable clusters represents three information subprocesses that differentially contribute to the overall confidence estimate. As the activity in these brain regions begins to covary, integration of multiple subprocesses occurs, which influences the probabilistic computation of a decision confidence estimate. Only the right-lateralized frontoparietal network correlated with metacognitive efficiency. This suggests that co-activation of confidence in a distributed frontoparietal network reflects metacognitive processing.

Many previous studies have shown that the quality of sensory information can influence decision confidence (Yeung and Summerfield, 2012; Boldt et al., 2017). Manipulation of stimulus strength and stimulus reliability has been shown to influence metacognitive performance (Boldt et al., 2017), which suggests that first-order and second-order decisions share similar processing mechanisms (Yeung and Summerfield, 2012). In line with this theory, empirical studies in primates and humans showed areas in the parietal cortex to encode both first-order decisions and second-order decisions during a post-decision wagering task (Kiani and Shadlen, 2009; Gherman and Philiastides, 2015). In our data, activation in the left and right IPL is significantly scaled with decision confidence. The left and right IPL were also part of a left-centered confidence co-activation cluster that also included the left SPL, basal forebrain, and Rolandic operculum. The left-centered parietal co-activation cluster may therefore represent confidence arising in decision-making areas (Kiani and Shadlen, 2009; Kiani et al., 2014). However, this co-activation cluster did not correlate with overall metacognitive efficiency, suggesting that metacognition depends on the integration of additional information.

An alternative theory suggests that different sources of information and processing mechanisms lead to first-order and second-order decisions respectively. In a heuristic model, first-order and second-order processes occur separately in a serial manner, with noise from the first-order decision and other internal states or information being evaluated in the second-order decision (Maniscalco and Lau, 2016; Fleming and Daw, 2017). Therefore, metacognitive performance is correlated to but independent of task performance. Disassociation between task performance and metacognitive performance has been identified in blindsight (Lau and Passingham, 2006), and psychiatric disorders (Rouault et al., 2018), but also across healthy individuals (Washburn et al., 2005; Fleming et al., 2010; Maniscalco and Lau, 2012; Bang and Fleming, 2018). Although behaviorally confidence ratings correlated with task accuracy in our data, we found subjects varied in their ability to accurately judge their task performance through confidence ratings. Recent research has tried to identify what types of information and the corresponding brain areas influence metacognitive performance independent of task performance. Action-specific information about first order-decisions in the premotor cortex contributes to metacognitive judgments independently of first-order decisions (Fleming and Lau, 2014; Wokke et al., 2020). Furthermore, functional connectivity between prefrontal and motor areas increased between the first-order response and metacognitive judgment (Wokke et al., 2020). Besides perceptual evidence, global internal states influence metacognitive performance. Global estimates of self-performance seem to be influenced by previous confidence estimates and choices on preceding trials, even during the absence of feedback (Benwell et al., 2019; Rouault et al., 2019). Arousal (Allen et al., 2016; Hauser et al., 2017) and attention (Rahnev et al., 2011) also correlate with disassociation between metacognitive performance and task performance. In human fMRI studies, neural correlates in rostrolateral PFC (Fleming and Dolan, 2012; Grimaldi et al., 2015), striatum (Hebart et al., 2014; Gherman and Philiastides, 2018), medial prefrontal cortex (De Martino et al., 2013), dorsal anterior cingulate cortex (Fleming and Dolan, 2012), and other frontoparietal regions (Vaccaro and Fleming, 2018) have been associated with metacognitive performance. The right-centered frontoparietal co-activation network (Figure 3A) in our results relates to previous findings, showing that confidence covaries across frontal and prefrontal regions along with the SEF and basal forebrain. We incorporated a ROI in the pgACC, which has previously been shown to track expected performance (Bang and Fleming, 2018). However, in our results, the pgACC did not correlate strongly with the other ROIs. Rather inter-subject variability in metacognitive efficiency correlated most strongly with decision confidence in the SEF.

We also identified a third co-activation cluster that consisted of regions in the left and right cerebellum. Traditionally, the cerebellum is thought to monitor and evaluate movement control by dually encoding internal predictions and sensory feedback, and reporting a subsequent error (Schlerf et al., 2012; Schmahmann, 2019). The cerebellum may likely have a similar role in other sensory modalities (Popa et al., 2014; Peterburs and Desmond, 2016) and cognitive processes (Stoodley et al., 2012). Error reporting, as well forward modeling, are elements that influence the degree of confidence. In this study, we are unable to differentiate whether the significant activity in the cerebellar co-activation cluster is related to confidence or other performance monitoring processes that contribute to decision confidence.

Our results provide a novel finding in humans in showing converging evidence that the supplementary eye fields are tightly coupled to the emergence of confidence and individuals’ metacognitive efficiency in our visual discrimination task. Previously, the SEF has only been shown to be involved in performance monitoring, including error monitoring and conflict-monitoring, in visual-oculomotor tasks in electrophysiological (Stuphorn et al., 2000; Emeric et al., 2010) and fMRI studies (Nachev et al., 2005). Although our study did not include saccade-based decisions, the SEF also correlated with high metacognitive efficiency in our results. A high metacognitive efficiency value (meta-d’/d’ > 1) has been suggested to indicate error monitoring, as the individual performs poorly on task discrimination yet is aware of their poor performance (Fleming and Daw, 2017). Higher metacognitive ability may therefore arise from faster, more robust signals in the SEF during a backward-masked discrimination task. Furthermore, we performed a control analysis that excluded trials containing saccades within 200 ms of target presentation (see the Supplementary Information for more detail) to exclude possible confounding effects of saccades on our results. The SEF also has been implicated in duration estimates of timing during uncertain tasks (Cui et al., 2009), however, a subsequent analysis of confidence across different inter-stimulus time intervals revealed no significant pattern in our results (see Supplementary Information). Therefore, our results suggest the activation of the SEF may be domain-general across different sensory modalities and metacognitive tasks. The SEF may serve as a hub for monitoring and evaluating local estimates of confidence arising from different types of information across distributed brain regions, leading to an overall global estimate of metacognitive ability.

It should be noted that the metacognitive efficiency ratios (meta-d’/d’) varied notably across subjects. A Pearson correlation performed between metacognitive efficiency (meta-d’/d’) and task discrimination sensitivity (d’) was trending towards significance (*p* = 0.056). We interpreted the non-significant correlation between metacognitive efficiency and discrimination sensitivity to signify independence in variability between the two measures across subjects. Because of the small subject number, the power of the study may not be strong enough to fully exclude a possible correlation between metacognitive efficiency (meta-d’/d’) and task discrimination (d’). In the case that the correlation would be significant, the value obtained for metacognitive efficiency would still take into account task performance bias, however, metacognitive performance would scale with task performance. We would interpret this to mean that the variability in metacognitive efficiency measures arises from variability in task performance across subjects rather than variability in both task and metacognitive performance. Concerning our findings, the frontoparietal network would still reflect activation patterns correlated with increasing metacognitive efficiency that is distinct from other processes leading to decision confidence. However, we would not be able to differentiate variability in metacognitive performance from the variability in task performance across subjects.

Our study is limited to retrospective confidence ratings in metacognitive perception. Metacognition can be reflected in different introspective processes such as decision uncertainty, reward expectancies, and error-monitoring (Vaccaro and Fleming, 2018). Domain-general and domain-specific regions have also been identified in visual metacognitive memory and visual perception tasks (McCurdy et al., 2013; Morales et al., 2018; Vaccaro and Fleming, 2018). Behavioral measures of metacognitive efficiency also correlate across different sensory modalities (Faivre et al., 2018; Beck et al., 2019), yet the neural correlates of decision confidence and metacognitive efficiency across sensory modalities remain elusive. Future studies are needed to understand how confidence estimates arise across different metacognitive tasks and whether decision confidence estimates covary in domain-general or domain-specific patterns across metacognitive task types.

In conclusion, we found activity correlated significantly with visual confidence in the supplementary eye field, bilateral basal forebrain, prefrontal, and parietal regions. We accounted for individual differences in metacognitive performance by including inter-subject variability in metacognitive efficiency in our fMRI analysis, which further confirmed the involvement of the SEF in visual metacognition. The SEF has been implicated in performance and error monitoring in non-human primate studies. Our results are the first to show the supplementary eye field’s role in metacognition in humans during visual perception. We implemented a novel approach, examining confidence dependent co-activation across brain regions that correlated significantly with decision confidence and found three segregable clusters: a right-centered frontoparietal cluster, a left-centered temporal-parietal cluster, and a cerebellar cluster. Only the right-centered co-activation cluster, which included the supplementary eye field, correlated with increased metacognitive efficiency. These findings suggest distinct information processes correlate with decision confidence, yet only the right-centered frontoparietal co-activation cluster reflects metacognitive ability. Our results are in line with the view that confidence estimates arise from the integration of multiple components. Distinct components are thought to be processed through different pathways, with only some processing pathways reflecting metacognitive ability.

## Data Availability Statement

The raw data supporting the conclusions of this article will be made available by the authors, without undue reservation.

## Ethics Statement

The study involving human participants was reviewed and approved by TUM School of Medicine at the Technical University of Munich. The participants provided their written informed consent to participate in this study.

## Author Contributions

AW and CS oversaw research. SG, CS, and AW designed research. SG and AR performed experiments. CJ, CD, SG, and AW analyzed data. CJ, CS, and AW interpreted results of experiments. CJ drafted manuscript, prepared figures, and edited and revised manuscript. All authors contributed to the article and approved the submitted version.

## Conflict of Interest

The authors declare that the research was conducted in the absence of any commercial or financial relationships that could be construed as a potential conflict of interest.

## Footnotes

^1^www.fil.ion.ucl.ac.uk/spm/
