# Supplementary material for: Segregated Co-activation Patterns in the Emergence of Decision Confidence During Visual Perception
Source: Front Syst Neurosci. 2020 Nov 10;14:557693. doi: 10.3389/fnsys.2020.557693 (PMC7683611; doi:10.3389/fnsys.2020.557693)
Supplement: Supplementary file 1 [file Table_1.DOCX]

Supplementary Material

# Supplementary Methods

*Eye blink and saccade removal:* EEG data was acquired through BrainVision Recorder software (Brain Products GmbH, Germany) from 63 scalp electrodes (EASYCAP GmbH, Germany). In the present study only EOG was evaluated for fixation monitoring. Electrodes were placed according to the 10/10 system. Eye movements were tracked via two electrodes positioned below the outer canthi of the eyes. Electrophysiological artifacts caused by eye movements were identified by applying a high-pass Butterworth filter with a 0.5 Hz cut off to DC trend-corrected data (see Hennighausen et al. 1993 for detrending method). Data was manually inspected and all trials containing artifacts were marked. A 64-component independent component analysis (ICA) was performed and all components containing eye blinks or saccades were selected (see Hipp and Siegel 2013 for methods). The time-domain was reconstructed from the selected components and corresponding trials containing eye movement, eye blink, and saccade artifacts were removed from subsequent behavioral and data analyses.

# Supplementary Data

*Testing for significance across interstimulus time intervals:* A previous study by Cui et al. (2009) reported an increase in SEF activity as a function of duration of inter-stimulus intervals (ISIs). To rule out that SEF activity and measures of confidence were influenced by duration of ISIs, a multi-way ANOVA was performed on the proportion of correct trials across ISI interval, confidence ratings and subject. No significance was observed for ISI intervals (F_1,180_  = 0.24, p = 0.656). The interaction between ISI and confidence (F_3, 180_ = 2.31 , p = 0.0775) was insignificant.

# Supplementary Figures and Tables

## Supplementary Figures


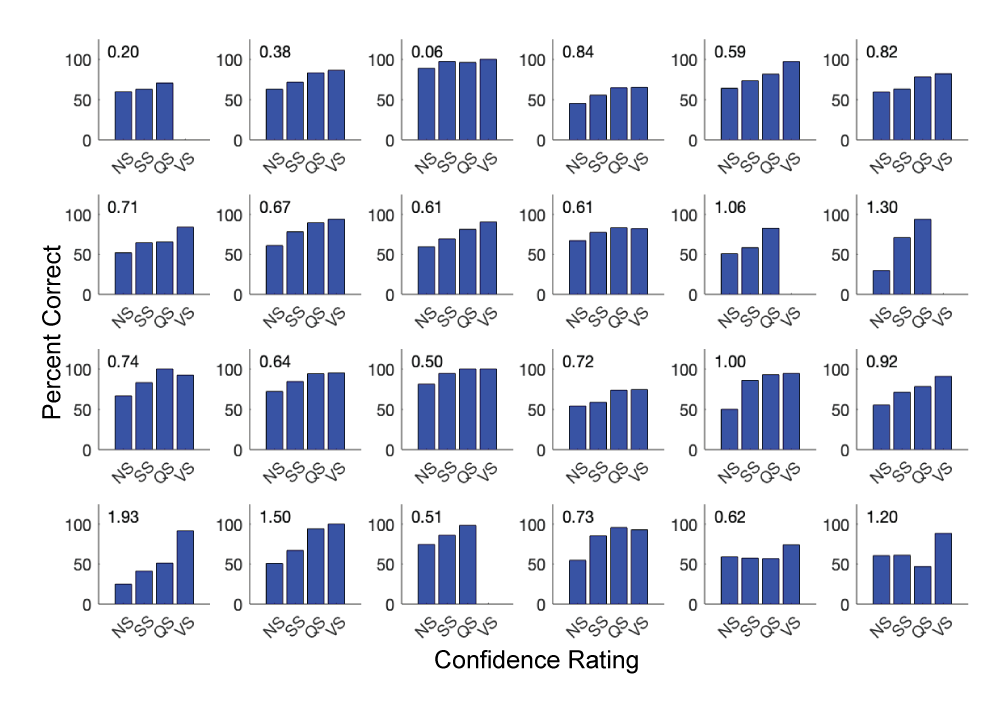


**Figure S1:** The percent correct trials for each confidence rating (NS = not sure, SS = slightly sure, QS = very sure, VS = very sure) for each subject showing inter-subject variability in overall confidence on the task. The individual metacognitive efficiency values (d’/meta-d’) are indicated in the top left corner of each subplot for each subject.


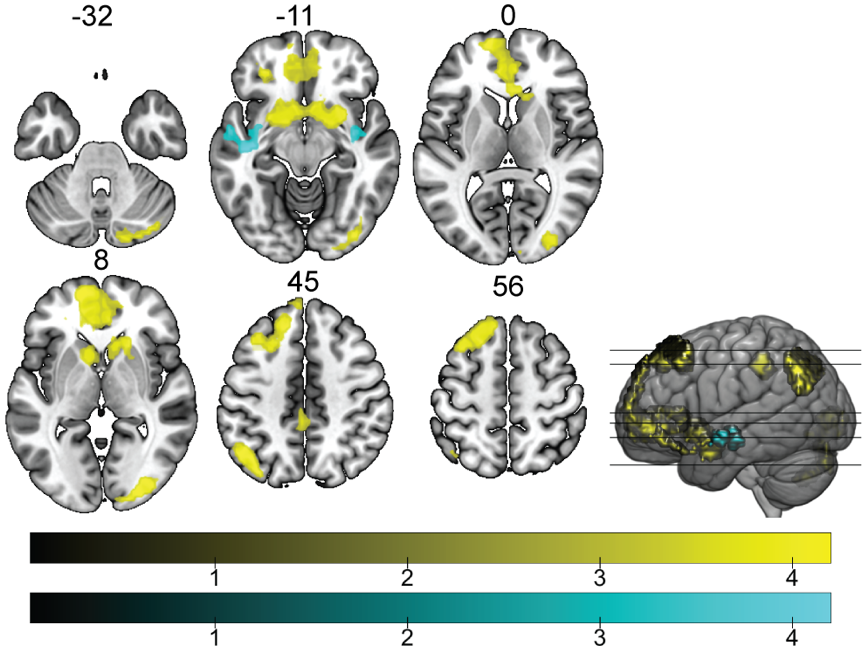


**Figure S2:** a) Significant clusters for the canonical HRF (yellow) with parametric modeling of confidence are overlaid with significant clusters (cyan) correlating to individual differences in metacognitive efficiency (both P_cc_ <0.05 at P_unc_ < 0.001 at voxel-level).


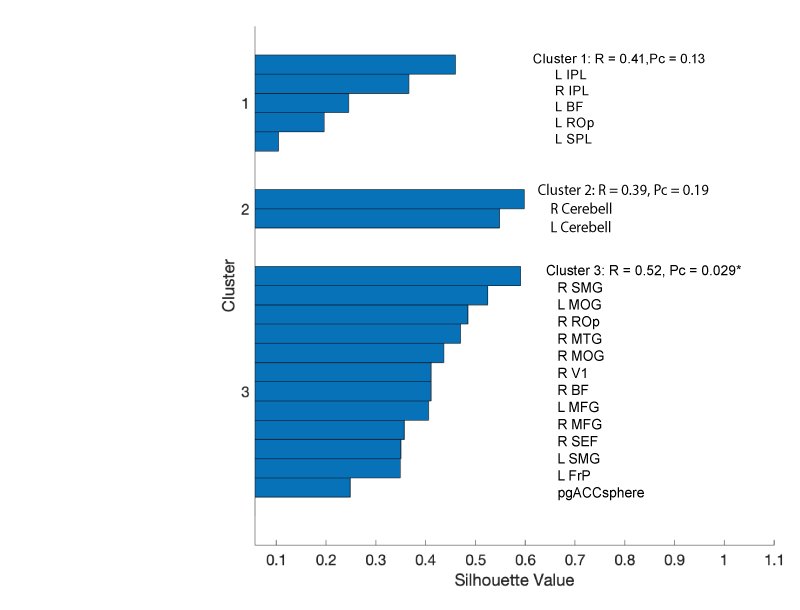


**Figure S3:** Silhouette plot for the cluster analysis. A) Silhouette values for the three clusters containing activated clusters from the temporal derivative contrast as well as pgACC sphere obtained from Bang & Fleming (2018) (MNI: -2/44/10). ROIs are categorized by attribution strength to the given cluster in descending order. Cluster centroids were subsequently correlated with subjects’ metacognitive efficiency values in Pearson correlations. P-values were Bonferroni-corrected for three comparisons. Only cluster 3 was significantly correlated with metacognitive efficiency.

**3.2 Supplementary Tables**

| **Table S1:** Peak voxels of significant clusters for canonical contrast (p_cc_ < 0.05 at P_unc_ < 0.001 voxel threshold) with increasing confidence | | |
| --- | --- | --- |
| Region | MNI Coordinates | Z-score |
| L ACC | -3/44/2 | 5.13 |
| L Superior Medial Gyrus | -15/59/2 | 4.93 |
| L IPL | -45/-64/-44 | 4.82 |
| R Inferior Occipital Gyrus | 33/-88/-1 | 4.78 |
| L Middle Frontal Gyrus | -21/29/62 | 4.56 |
| Superior Parietal Lobe | 0/-40/44 | 3.67 |

| **Table S2:** Peak voxels of significant clusters for canonical contrast (p_cc_ < 0.05 at P_unc_ < 0.001 voxel threshold) with increasing confidence correlating to increase in metacognitive efficiency | | |
| --- | --- | --- |
| Region | MNI Coordinates | Z-score |
| R Middle temporal gyrus | 48/2/-25 | 4.62 |
| L Middle temporal gyrus | -42/-16/-16 | 4.39 |

**4. SI References:**

1. Cui, Xu, Chess Stetson, P. Read Montague, and David M. Eagleman. 2009. “Ready…Go: Amplitude of the FMRI Signal Encodes Expectation of Cue Arrival Time.” Edited by James Ashe. *PLoS Biology* 7 (8): e1000167. https://doi.org/10.1371/journal.pbio.1000167.
2. Hennighausen, Erwin, Martin Heil, and Frank Rösler. 1993. “A Correction Method for DC Drift Artifacts.” *Electroencephalography and Clinical Neurophysiology* 86 (3): 199–204. https://doi.org/10.1016/0013-4694(93)90008-J.
3. Hipp, Joerg F., and Markus Siegel. 2013. “Dissociating Neuronal Gamma-Band Activity from Cranial and Ocular Muscle Activity in EEG.” *Frontiers in Human Neuroscience* https://doi.org/10.3389/fnhum.2013.00338.
